# Cultivar selection and processing methods are crucial tools for tailoring the physico-chemical properties and functionality of pea (*Pisum sativum* L.) protein ingredients

**DOI:** 10.1016/j.crfs.2025.101218

**Published:** 2025-10-10

**Authors:** Jason D. Foley, Tomasz P. Czaja, Maria M. Rieckmann, Malbor Dervishi, Søren B. Engelsen, Kristian H. Laursen, Andrea Bononad-Olmo, Wender L.P. Bredie, Vibeke Orlien

**Affiliations:** a Department of Food Science, University of Copenhagen, Rolighedsvej 26, 1958, Frederiksberg C, Denmark; b Department of Plant and Environmental Sciences, University of Copenhagen, Thorvaldsensvej 40, 1871, Frederiksberg C, Denmark

**Keywords:** Pea protein, Concentrate, Isolate, Foamability, Physico-chemical characteristics, pH effects

## Abstract

Ten cultivars of pea (Akooma, Astronaute, Bagoo, Greenway, Ingrid, Kaplan, Karacter, Manager, Orchestra, and Skol) were grown to advance the understanding of the functional diversity, sensory quality, and applicability of pea proteins. The pea seeds were processed into two protein-rich ingredients: concentrates (mean protein content of 48.3 %) by dry-fractionation and isolates by wet extraction (mean protein content of 80 %). The isolates were characterized by denatured protein particles and high natural pH (6.7–8.2) compared to the concentrates containing native protein and lower natural pH (5.4–5.9), including other constituents such as saponins and fibers. The differences in compositional characteristics did not affect physico-chemical characteristics, but greatly impacted ingredients’ techno-functional properties, and in turn their food applicability. Water and oil holding capacities were higher in the isolates, 7.54 g water/g powder and 3.54 g oil/g powder, compared to concentrates, 2.16 g water/g powder and 1.63 g oil/g powder, highlighting important differences in the ingredient interaction with water and oil. In contrast, the concentrates exhibited higher protein solubility, up to 65 % in neutral and up to 81 % in alkaline systems, outperforming the isolates with protein solubilities near 15 %. A higher foaming ability of concentrates (40–70 %) in comparison to isolates (30–60 %) was found at their natural pH. Smaller particle size enhanced foam forming ability of concentrates, while the aggregates in isolates may interfere with the foam formation mechanism. Concentrates were perceived as sweet, umami, bitter, and astringency tasting. The flavors, green pea, yellow pea, nutty, and bitter after taste, differed significantly between the different cultivars.

## Highlights

• 10 different pea cultivars showed clear differences in application potential.
• Pea protein concentrates had the highest solubility and foam capacity.
• Pea protein isolates had the highest water and oil holding capacity.

## Graphical abstract

**Figure:**
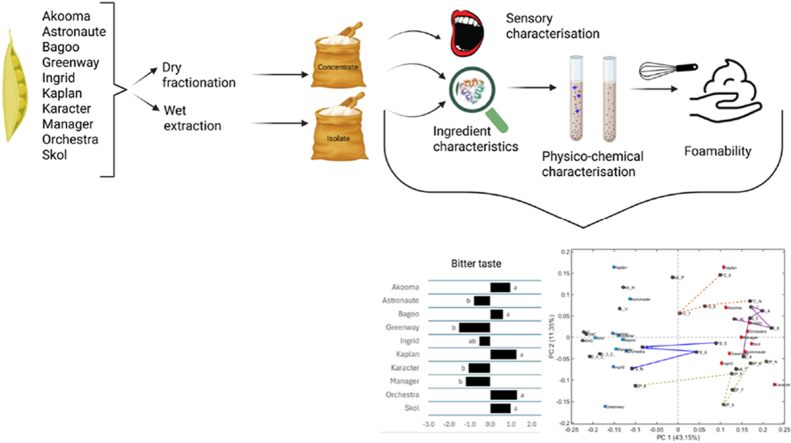

## 1 Introduction

Legume breeding has mainly targeted high-yielding and disease-resistant cultivars to meet the requirements for animal feed and human consumption. The green transition in agriculture and food production has provided new complex targets for plant breeding, including quality traits based on raw materials and derived ingredients made from legumes (Khakimov et al., 2025). To advance the quality of plant-based food products we need to understand what the most important quality requirements are. Both the cultivar and post-processing methods are known to influence the performance of derived protein ingredients. Pea (*Pisum sativum* L.) has attracted attention due to its ability to grow in colder climates (compared to soy cultivation) and high potential to substitute soy proteins. Commercial pea proteins (PP) are extracted from pea seeds by dry fractionation or wet extraction, resulting in two types of ingredients: concentrates or isolates. Pea concentrates have a low protein content of 50–75 % (dry basis) but their proteins often remain in native state. In contrast, the high purity isolates have a protein content above 75 % (Amin et al., 2022). Pea isolates are produced using alkali extraction, isoelectric precipitation, and spray drying (Schmitt et al., 2021). These harsh extraction processes often affect protein conformation, leading to denaturation and aggregation of proteins to various degrees, thereby influencing their functional performances in food formulations (Burger et al., 2022). Numerous reviews have documented research about how PPs can deliver a large array of technological functions essential for food products, e.g., solubility, gelling, emulsifying, foaming, water, and oil absorption properties (Grossmann and McClements, 2023b; Ge et al., 2020; Lam et al., 2018; Lu et al., 2019; Wu et al., 2023). It is thus largely demonstrated that the techno-functionality of PPs depends on the cultivar, growth conditions and climate as well as the type of fraction. Challenges with discrepancies and inconsistent findings across various investigations make it difficult to determine the precise requirement for a specific functional property. Hence, to date, using PP ingredients still poses challenges in the development of pea-based foods due to significant differences in functional properties (Ge et al., 2020; Lam et al., 2018). To improve the applicability of PP ingredients in the food industry, more systematic and comprehensive studies are required for relating the physico-chemical characteristics with the functional properties across a range of different cultivars. Furthermore, little is known about the variation in sensory quality of PP ingredients in relation to pea cultivars. Sensory evaluation of the PP ingredients can provide insights into desirable and less desirable sensory properties and help to bridge the gap between techno-functional performance and perceived quality. This knowledge is valuable for end-product development and can guide relevant cultivar selection to better fit with consumer acceptance.

Among the functional properties, foaming is especially relevant due to its role in conferring desirable textural and sensory attributes to food products, such as bread, whipped topping, ice cream, and carbonated drinks. Foam is a colloidal system with air bubbles dispersed in the continuous phase of liquid (Damodaran, 2005). Applying PP ingredients as a foaming agent faces inherent limitations due to their structural heterogeneity and relatively low solubility, which affect the adsorption ability at the air-water interface and capacity to form and stabilize foams. For example, it has been reported that the foam properties of PPs are largely determined by the changes in the protein conformation (Chao and Aluko, 2018; Hall and Moraru, 2021).

This study reports the investigation of 10 different cultivars of peas processed by two different processing methods, dry fractionation and wet extraction, into a total of 20 PP ingredients. The PP concentrates and isolates were characterized, and the physico-chemical and functional properties were evaluated at different pHs relevant to food products. This broad-scale study contributes to a deeper understanding of inter-cultivar differences in relation to the production of protein-rich ingredients and informs the selection of suitable pea cultivars and processing method for specific food applications by both farmers and food producers. In addition, to address these scientific and applied gaps, the results will provide the scientific community with more data to contribute to the in-depth understanding of the functionality of plant proteins.

## 2 Materials and methods

### 2.1 Materials

**Fig. 1:**
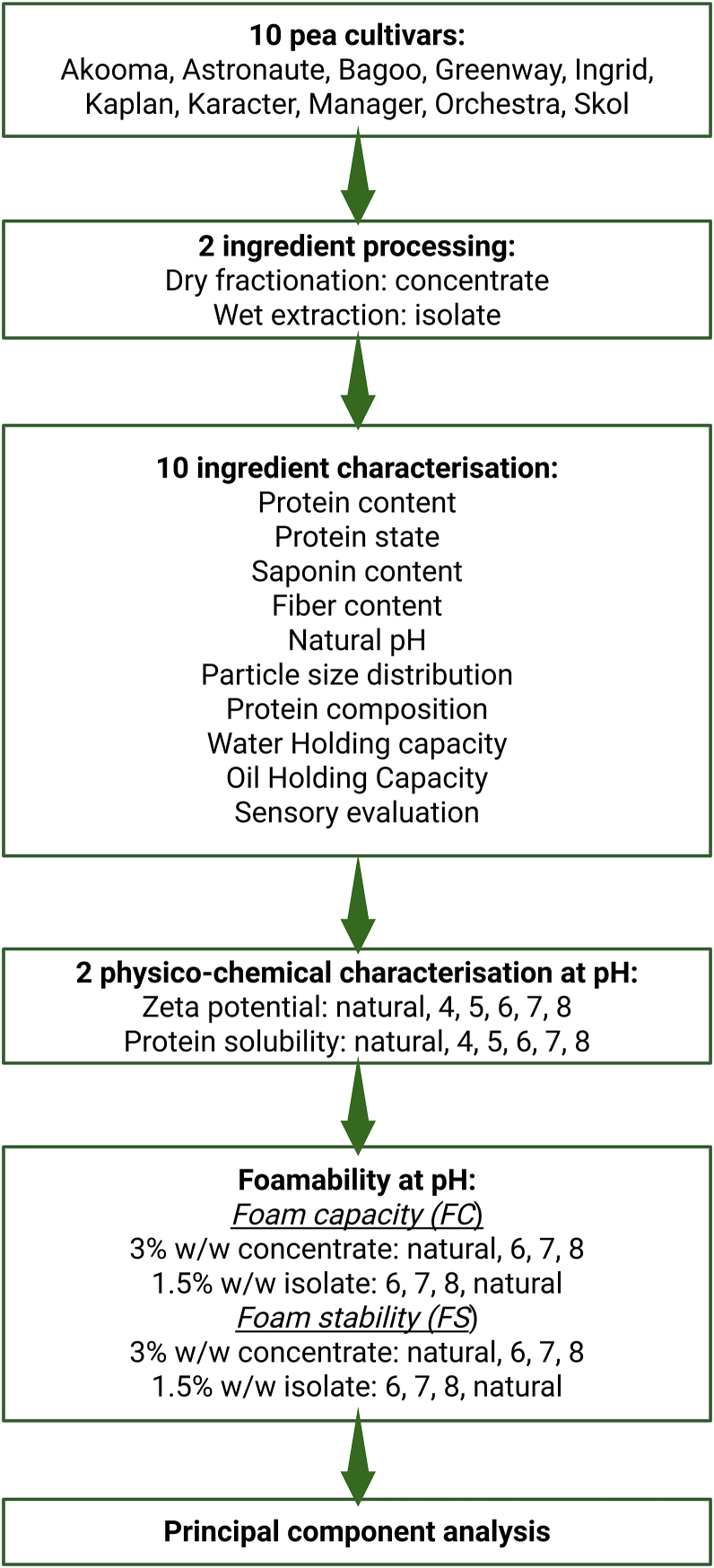
Overview of the experimental design; 10 pea cultivars were processed into two types of ingredients, PP concentrates (dry fractionation) and PP isolates (alkaline extraction). Ingredient powders were characterized, and the physico-chemical characteristics were assessed at pH 4–8. Foamability was established at the pH values: 6, 7, and 8 as well as natural pH. Created in https://BioRender.com.

Ten cultivars of field peas (*Pisum sativum* L.), Akooma, Astronaute, Bagoo, Greenway, Ingrid, Kaplan, Karacter, Manager, Orchestra, and Skol, were grown in Hammel (Denmark) in 2022 on a coarse sandy clay soil. Sowing was conducted on the 12th of May and the peas were grown according to the principles of organic farming (EU Regulation, 2018/848). No fertilizers were applied. At maturity, ultimo August/primo September, peas were harvested and stored in 20 kg aerated bags. The peas were then cleaned, dried, dehulled and subsequently processed into protein concentrates or isolates. Pea protein concentrates were obtained by air fractionation (Danish Technological Institute, Skejby, Denmark). In short, dehulled peas were milled into flour, following a spiral air stream to separate the protein fraction. Pea protein isolates were obtained by wet extraction (Cosucra-Groupe Warcoing, Pecq, Belgium). In short, the process was: dehulled peas (NSP Grain Cleaner, Chopin Technologies, Cedex, France), dry milling, solubilization (pH about 8), decantation, thermal treatment (pasteurization at 72 °C for 20–40 s), concentration (pH 4.7), neutralization, thermal treatment (in boiling water for 15 min) and lyophilization (Beta 1–8 LSCplus, Christ, Osterode am Harz, Germany). Concentrate and isolate processing were performed at pilot scale resembling industrial processing. Fig. 1 shows the overview of samples and related analyses as presented in the following sections.

### 2.2 Protein content

The total nitrogen content of the PP concentrates and isolates was analyzed using an elemental analyzer (EA) (PYRO Cube Elemental Analyzer, Elementar, Hanau, Germany) coupled to an isotope ratio mass spectrometer (IRMS) (Iso-prime100, Elementar, Manchester, UK) as described by Novak et al. (2019). An amount of 1.5 mg of sample powder was transferred into a tin capsule and analyzed in the EA combustion mode. The nitrogen content was calculated from calibration curves of acetanilide (Sigma Aldrich). The analytical error was determined from the standard deviation of repeatedly measured acetanilide samples and was 0.04 %. A conversion factor of 6.25 was used to calculate the protein content based on the measured nitrogen values (Mariotti et al., 2008). For protein analysis 20 % of samples were analyzed in duplicate and the standard deviation was 0.02–1.5 for concentrates and 0.02–0.2 for isolates.

### 2.3 Differential scanning calorimetry (DSC)

The thermal properties of the PP ingredients were investigated with a differential scanning calorimeter (820, Mettler Toledo, Schwerzenbach, Switzerland), which is based on the heat flux principle. The heat flow and temperature were calibrated with indium (*T*_*m*_ = 156.6 °C, Δ*Hf*_*us*_ = 28.5 J/g) and zinc (*T*_*m*_ = 419.5 °C, Δ*H*_*fus*_ = 107.5 J/g) as standards. Approximately 1 g of the PP was hydrated to a concentration of 50 %. An aliquot (30–40 mg) of this suspension was transferred to a 40 μL aluminum DSC crucible (ME 27331) and hermetically sealed. An empty crucible was used as a reference. The samples were scanned from 25 to 110 °C with the heating scan rate of 10 °C/min. The peak temperature of the endothermic peaks was obtained by the built-in software, STAR^e^, corresponding to the starch gelation temperature (1^st^ peak) and protein denaturation temperature (2^nd^ peak).

### 2.4 Saponin content

A total of 100 mg of dried material was accurately weighed and transferred into a 2 mL Eppendorf tube (Safe lock tube). Extraction was performed using 1 mL of 85 % ethanol (96 % v/v, CWR Chemicals) and facilitated by an ultrasonic bath (5510, Branson) for 60 min. To ensure thorough extraction, samples were manually shaken every 10 min. Following extraction, samples were centrifuged at 14680 rpm/20238 g (Centrifuge 5424, Eppendorf) for 5 min. Prior to measurement, samples were diluted by a factor of 20, stored at 4 °C and filtered through a 0.2 μm Durapore 96-well plate membrane (Millipore, Ireland). Details of LC-qToF-MS/MS data according to (Trinh et al., 2024) and detailed description of the LC-MS procedure can be found in Appendix A. After analysis, the concentration of the target saponin was determined through relative quantification using a standard calibration curve of soyasaponin-Ba (PhyProof, Germany) as a reference, which measured through the same LC-qToF-MS/MS protocol.

### 2.5 Fiber content

The content of total, soluble, and insoluble dietary fibers was determined according to the Megazyme (Neogen Corporation, US) procedures provided with the test kit (Approved Methods 32–07.01, AACC, 2000). It is noted that the very low content of fiber in the PP isolates resulted in negative values of soluble fiber (due to the calculation involved), thus is not reported.

### 2.6 Natural pH

Each of the PP ingredients was suspended in tap water at a 1 % (w/w) powder concentration, thus at natural condition, and stirred using a magnetic stirrer at 600 rpm for 30 min at room temperature. The natural pH of each PP ingredient suspension was measured with a benchtop pH meter (Mettler Toledo FiveEasy F20 Greifensee, Switzerland).

### 2.7 Particle size distribution

A Malvern Mastersizer 3000 (Malvern instrument Ltd, Malvern, England) was used to measure the particle size distribution of the PP ingredients. Each of the PP ingredients was suspended in Milli-Q water at a 1 % (w/w) powder concentration and stirred using a magnetic stirrer at 600 rpm for overnight hydration at room temperature. The refractive indices of the dispersed phase and the deionized water were set at 1.45 and 1.33, respectively. Each sample was added in the connected Hydro 2000S wet dispersion unit until reaching the obscuration between 5 % and 12 %. The volume average diameter, D[4,3], and surface average diameter, D[3,2], were used to describe the size of particles. Measurements were conducted in duplicate at room temperature, with each sample run five times to ensure accuracy.

### 2.8 Protein composition

#### 2.8.1 Electrophoresis (SDS-PAGE)

The gel electrophoresis was performed to determine the protein fraction patterns according to (Sharan et al., 2022), with some modifications. In brief, 20 mg PP powder (concentrates and isolates) was dissolved in 600 μL of 0.1 M Tris-HCl buffer (containing 5 % SDS, pH 8.0). The protein suspension was mixed using a Mixer mill (MM400, Retsch, Hann, Germany) at a frequency of 30 Hz for 10 min (5 min as a cycle, two cycles) to enhance protein solubilization. After this, the mixture was heated at 80 °C and 350 rpm for 10 min in a thermos mixer (Provocell, Esco Micro Pte. Ltd, Singapore) followed by centrifugation (20000 g, 5 min). The protein concentration of the supernatant was adjusted to approximately 1.54 mg/mL. Under reduced conditions, an aliquot of 65 μL of protein supernatant was mixed with 25 μL of 4 × lithium dodecyl sulfate (LDS) buffer and 10 μL of 1 M reducing agent dithiothreitol (DTT), and the mixture was heated at 80 °C for 10 min. The non-reduced samples for gel electrophoresis were prepared the same as above, though 10 μL of Milli-Q water was added to replace DTT to reach a total volume of 100 μL. The reduced and non-reduced samples were heated at 80 °C for 10 min. Each of the prepared samples (10 μL) and SDS-PAGE molecular mass standards (3 μL) were loaded onto a 12 % NuPAGE Bis-Tris gel. The running buffer was prepared by diluting 20 × Tris/MOPS SDS running buffer. Gel electrophoresis was run at 120 V for 20 min and then 180 V to the end. Gels were stained overnight with Coomassie brilliant blue solution and then de-stained using Milli-Q water. Protein bands were scanned using an Epson Perfection V750 pro scanner (Nagano, Japan). In the SDS–PAGE analysis, the same total protein concentration (1 mg/mL) is loaded on each lane, hence the PAGE result shows the relative distribution of the individual solubilized proteins in a comparable mode. The band identification and relative quantification on the 11S/7S ratio was carried out by GelAnalyzer software (GelAnalyzer 23.1.1, www.gelanalyzer.com) by the 1D analysis mode. The 11S/7S ratio was calculated using the volumes of the bands identified only as legumin and vicilin.

#### 2.8.2 Amino acid analysis

Analysis of the amino acid (AA) composition of concentrates and isolates was performed by UPLC-MS as described by Dahl-Lassen et al. (2018) and la Cour et al. (2019). Approximately 20 mg of sample was used for each duplicate analysis. Acid hydrolysis was conducted using 6 M HCl with 0.1 % w/v phenol at 110 °C for 24 h. After hydrolysis, sample neutralization was performed with 6 M sodium hydroxide. An oxidation with performic acid for 1 h at room temperature was performed prior to hydrolysis for analysis of sulfur-containing AAs (cysteine and methionine). In order to quench the reaction, solid sodium metabisulfite was added. Hydrolysis was subsequently proceeded as described above. Regarding tryptophan, 20 mg of sample was mixed with 50 mg ascorbic acid and 3 ml 4 M LiOH and was then hydrolyzed at 110 °C for 20 h. Sample neutralization was done with 2 ml 6 M HCl. Amino acids were then derivatized using the analytical grade AccQ-Tag kit (Waters, Millford, MA, USA). Separation of AAs was done using a Waters UPLC system (Waters, Millford, MA, USA) with a Cortecs UPLC C18 column (1.6 μm particle size, 2.1 × 150 mm) with a VanGuard Cor-tecs UPLC C18 guard column (1.6 μm particle size, 2.1 × 5 mm) (Waters, Millford, MA, USA). The derivatized AAs were detected using a Waters QDa single quadrupole mass detector. The following AAs were detected: alanine, arginine, glycine, histidine, isoleucine, leucine, lysine, phenylalanine, proline, serine, threonine, tyrosine, valine, aspartic acid, glutamic acid, cysteic acid, methionine sulfone, and tryptophan. The certified reference materials 1849a (Infant/Adult Nutritional Formula I) and 3234 (Soy flour) from National Institute of Standards and Technology (NIST) were used for quality control.

### 2.9 Zeta potential

The ζ-potential of the PP ingredients was measured using Zetasizer Nano SZ (Malvern, Worcestershire, UK) based on (Patra et al., 2022). Each of the PP ingredients was suspended in Milli-Q water at a 1 % (w/w) protein concentration and stirred using a magnetic stirrer at 600 rpm for overnight hydration at room temperature. pH was adjusted by 100 mM HCl or 100 mM NaOH prior to centrifugation and was rechecked before measurement, with further adjustments made if necessary. PP suspensions were not adjusted for measurement at neutral pH. The PP suspensions were centrifuged at 4500 g for 10 min at room temperature (to remove undissolved proteins and particles) and the supernatants were loaded in a special capillary cuvette with two electrodes for the ζ-potential determination. The electrophoretic mobility (velocity) of a particle was measured by applying an external electric field and correlated using the Smoluchowski model. The velocity is transformed to a zeta potential value using the Henry equation. Refractive indices were set to 1.45 for the protein and 1.33 for the water phase. Each measurement included 5 sets of 30 runs, conducted in duplicate at 25 °C.

### 2.10 Water (WHC) and oil (OHC) holding capacity

Both water holding capacity (WHC) and oil holding capacity (OHC) analyses were conducted in triplicate for each PP ingredient. For WHC, a 15 mL centrifuge tube was weighed (M), and 0.3 g of powder was added to the tube with the weight recorded as M1. 7 ml of water was then pipetted into the tube, and the mixture was vortexed until the powder was completely suspended in water. The tube was incubated in a water bath at 30 °C for 60 min, followed by cooling at room temperature for 10 min. Subsequently, the sample was centrifuged at 2000×*g* for 15 min, and the supernatant was poured off. The tube, now containing the wet pellet, was weighed, and the weight was recorded as M2.

Water holding capacity (WHC) was calculated as:

$$\text{WHC}\;\left(g\;\text{water}/g\;\text{powder}\right)=M2-\frac{M1+M}{M1}$$

Where: M = weight of the empty centrifuge tube, M1 = weight of the powder and tube, M2 = weight of the tube with the wet pellet after centrifugation.

For OHC, 0.3 g of powder was weighed into a 15 mL centrifuge tube (M), and 3 g of oil was added, with the weight recorded as M1. The mixture was vortexed to ensure thorough mixing, then incubated in a water bath at 30 °C for 60 min, followed by cooling at room temperature for 10 min. The sample was centrifuged at 2000×*g* for 15 min, and the free oil (supernatant) was poured into a tared beaker, with the weight of the collected oil recorded as M2.

Oil holding capacity (OHC) was calculated as:

$$\text{OHC}\;\left(g\;\text{oil}/g\;\text{powder}\right)=M1-\frac{M2}{M}$$

Where: M = weight of the powder, M1 = initial weight of the oil, M2 = weight of the free oil after centrifugation.

WHC and OHC were both measured in triplicate.

### 2.11 Sensory evaluation

Raw PP concentrates of all 10 pea cultivars were evaluated by a trained sensory panel (n = 9) at the Department of Food Science, University of Copenhagen. Sensory evaluation was not performed on PP isolates due to limitation in amount. A descriptive sensory profiling was performed, where the panel over three sessions of 2 h developed a sensory vocabulary and trained on the intensity scales (15 cm) and references (Appendix A, Table S1). The samples were assessed in sensory booths and involved tasting of aliquots of 1 g raw pea concentrate powder in black plastic containers at 22 °C. Samples were labelled by a random 3-digit code and tasted using a stainless-steel teaspoon, which was cleaned between tastings. The samples were presented in a randomized balanced block design with five samples per block. Over two test days, three blocks per day were evaluated with 10 min break in between, corresponding to 3 replicated product evaluations by the panel.

In a second sensory evaluation, 4 trained assessors evaluated by a similar procedure the raw and heat-treated (165 °C for 5 min) PP concentrates. In this evaluation only bitter taste, beany flavor and roasted flavor were measured in two replicates.

### 2.12 Protein solubility

The protein solubility was determined according to (Feyzi et al., 2018), with slight modifications. Briefly, the PP ingredient suspensions (as described in 2.9.) were centrifuged at 9000 g for 20 min at room temperature. After centrifugation, the volume of the supernatant was recorded for calculation. The protein concentration in the supernatant was determined using a PierceTM bicinchoninic acid (BCA) assay kit (Thermo Fisher Scientific, Waltham, Massachusetts, USA). Protein solubility was calculated as the percentage of protein content in the supernatant to the protein content in the powder.

### 2.13 Foamability

The foaming properties of the PP ingredients were determined by mechanical foaming. A powder dispersion was prepared for foaming analysis, with concentrations of 3 % (w/v) for protein concentrates and 1.5 % (w/v) for protein isolates in order to have approximately the same protein levels. The powders were hydrated overnight, with pH adjustments made as necessary. The pH of each dispersion was measured before foaming analysis. For foaming capacity and stability measurements, mechanical foaming was conducted using an Ultra Turrax (UT) homogenizer (IKA T25, IKA, Ger-many). A rinsing step was conducted between samples to avoid cross-contamination. Each sample was prepared by placing 20 ml of the 3 % or 1.5 % powder dispersion in a 50 ml Falcon tube. The UT was set to a speed of 10 (10,000 rpm) for 35 s to foam the solution. During homogenization, the UT arm was fully submerged to ensure consistent foam formation. Following homogenization, the foamed solution was immediately transferred to a 50 ml graduated cylinder. The foam volume was recorded at 1 min (for foam capacity) and again at 30 min (for foam stability). Each sample was measured in duplicate to ensure reliability.

Foam capacity was calculated as foam overrun:

$$\text{FC}\;\left(\%\right)=\frac{\text{foam}\;\text{volume}\;\text{at}\;1\;\min}{\text{initial}\;\text{liquid}\;\text{volume}}\times 100$$

Foam stability was calculated as:

$$\text{FS}\;\left(\%\right)=\frac{\text{foam}\;\text{volume}\;\text{at}\;30\;\min}{\text{foam}\;\text{volume}\;\text{at}\;1\;\min}\times 100$$

### 2.14 Statistical analysis

Descriptive sensory profiling data were analyzed by a 2-way general linear mixed model ANOVA with assessors a random factor. Attributes with a significant (p < 0.05) sample effect were further analyzed by Tuckey HSD post hoc test. A 2-way generalised linear ANOVA was performed on the sensory data from the comparison of raw and heated PP concentrates (IBM SPSS v29.0.2.0). Pearson correlation coefficients were calculated to evaluate the linear relationships between variables based on data matrices obtained from various measurements. Computed correlation values were visualized using heatmaps generated in MATLAB 2022b. Separate correlation analyses were conducted for pea isolate and pea concentrate datasets to facilitate the identification of distinct correlation patterns within each sample type. Principal component analysis (PCA) was carried out using PLS-Toolbox 9.0 in MATLAB environment to determine relationships among the PP ingredient characteristics and foam properties. Results of total saponin, soluble and insoluble fiber were excluded from the PCA as the PP isolates did not contain those compounds.

## 3 Results and discussion

### 3.1 Characterization and physico-chemical characteristics

#### 3.1.1 Protein content

The legume seed proteins are metabolically inactive or storage proteins and constitute the major proteins of the seeds. The total protein content of the 10 de-hulled pea cultivars ranged between 22.1 % and 27.3 % (Appendix A, Table S2). In general, large variations in protein content in peas have been published and it is well known to be an effect of cultivars, field locations (e.g., soil fertility), growing year (e.g., wet or dry), farming practices (e.g., fertilization), and time of harvest. Differences in protein content based on cultivar have been reported previously: 14.95 %–22.44 % (10 varieties) (Guleria et al., 2009), 22.3 %–31.7 % (6 varieties) (Barac et al., 2010), 16.3 %–20.4 % (8 varieties) (Gupta & Mahajan, 2010), 21.3 %–28.4 % (6 varieties) (Wang et al., 2010), 17.8 %–23.5 % (37 varieties) (Choi et al., 2022). To improve food applicability, the pea seeds are specifically processed into two protein-rich ingredients as either concentrates or isolates. As seen in Table 1, the industrial-like processing (used in the present study) into PP concentrates and PP isolates increased the protein content, with the isolates having considerably higher protein content. A notable difference in protein content existed among the PP concentrates and isolates with a larger variability for the concentrates (mean protein content of 48.3 % ± 3.7), compared to the isolates (mean protein content of 80 % ± 1.5 %). Variability in the protein content of wet extracted (laboratory scale alkali-isoelectric extraction) PP isolates from different cultivars has been reported; 83 %–89 % (Barac et al., 2010), 91 %–95 % (Shevkani et al., 2015), 81 %–89 % (Stone et al., 2015a), 83 %–87 % (Stone et al., 2015b), and 90 %–93 % (Lam et al., 2017). The inherent protein content variation in the pea seeds is also found in the PP ingredients. However, no high correlations between the protein content in the seeds with the protein content in the concentrates or the isolates were found. Nevertheless, the protein content of the concentrates correlated with the protein content of the isolates, where Bagoo and Manager have low protein content in both concentrate and isolate, and Kaplan has high protein content in both concentrate and isolate. The protein content of other commercial PP protein isolates ranged 77 % (Shand et al., 2007), 72 % (Pelgrom et al., 2015), 80 %–85 % (Jakobson et al., 2023), and 76 %–84 % (Sun et al., 2023). Considering the total protein content, it should be noted that published literature values may vary due to differences in the applied analytical method and, especially the specific N-conversion factor used, wherefore direct comparisons should be made with care. For example, Ebert et al. (2020) showed that the specific protein contents (68.6 %–71.1 %) based on pea genus-specific conversion factor of 5.36 were 12–17 % lower than their crude protein contents (79.6 %–82.9 %) calculated using the conventional conversion factor of 6.25. Nevertheless, the N-factor 6.25 was used in most of the above cited studies and only the study by (Pelgrom et al., 2015) used 5.4 to calculate protein content. In the present study an N-factor of 6.25 was used to facilitate a quantitative comparison of the purity of the PP ingredients based on the applied fractionation and extraction process and their impact on PP functionality. The state of the proteins and the thermal behavior of the PP ingredients were determined by DSC (results not shown) and revealed significant differences between the concentrates and the isolates. The starch gelation temperatures and protein denaturation temperatures were reasonably similar between the different PP concentrates, in the range 65–67 °C and 83–88 °C, respectively (Table 1). The two endothermic peaks were absent in the thermograms of the PP isolates, indicating that protein denaturation had previously occurred during the PP ingredient processing.

**Table 1:** PP ingredient characterization.

Cultivar	Protein content^a^ (% d.m.)	Starch gelation temperature (°C)	Protein denaturation temperature (°C)	Saponin^b^ (mg/kg d.m.)	Soluble fiber (% d.m.)	Insoluble Fiber (% d.m.)	Natural pH	D[4,3] (μm)	D[3,2] (μm)
***Concentrate***									
Akooma	50.8	67.0 ± 0.2	88.0 ± 0.1	90.3	1.4	14.7	5.6	30.4 ± 0.61	17.0 ± 0.42
Astronaute	41.3	65.3 ± 0.3	86.9 ± 0.1	106.4	1.2	10.7	5.6	28.0 ± 0.52	14.7 ± 1.2
Bagoo	45.8	67.4 ± 0.3	86.2 ± 0.4	116.1	1.3	12.3	5.5	26.9 ± 0.50	15.5 ± 0.41
Greenway	46.8	66.8 ± 0.0	84.2 ± 0.1	60.2	1.1	14.4	5.9	27.7 ± 0.57	15.8 ± 0.53
Ingrid	47.0	65.4 ± 0.1	87.0 ± 0.1	107.9	1.4	13.6	5.8	32.1 ± 0.81	16.0 ± 0.52
Kaplan	54.4	66.9 ± 0.1	87.0 ± 0.4	196.6	2.2	11.8	5.5	25.1 ± 0.66	14.6 ± 0.36
Karacter	50.1	66.5 ± 0.5	87.3 ± 0.4	120.7	0.9	13.2	5.4	25.3 ± 0.39	15.1 ± 0.38
Manager	45.7	65.1 ± 0.4	86.0 ± 0.1	158.1	1.9	14.8	5.4	27.0 ± 0.63	15.4 ± 0.43
Orchestra	51.5	65.5 ± 0.1	83.4 ± 0.1	114.4	1.4	14.3	5.8	27.0 ± 2.29	13.8 ± 1.0
Skol	49.6	65.4 ± 0.0	86.8 ± 0.0	138.2	1.1	14.6	5.8	28.4 ± 1.2	16.4 ± 0.43
*Mean*	48.3 ± 3.7	66.1 ± 0.9	86.3 ± 1.4	120.9 ± 37.2	1.4 ± 0.4	13.4 ± 1.4	5.6 ± 0.2	27.8 ± 2.2	15.4 ± 0.9
***Isolate***									
Akooma	80.5			–			7.8	53.2 ± 3.2	22.2 ± 0.53
Astronaute	80.2			–			7.8	58.7 ± 3.7	26.9 ± 0.74
Bagoo	78.8			–			8.1	62.5 ± 4.3	27.9 ± 0.82
Greenway	78.4			–			7.7	72.7 ± 5.5	30.0 ± 1.2
Ingrid	78.4			–			8.2	55.6 ± 3.4	21.9 ± 0.40
Kaplan	83.3			–			7.1	50.8 ± 3.4	21.6 ± 0.62
Karacter	79.7			–			7.5	43.6 ± 3.5	17.9 ± 0.9
Manager	78.8			–			6.7	78.5 ± 3.7	36.5 ± 0.74
Orchestra	81.1			–			7.4	57.5 ± 3.7	22.9 ± 0.39
Skol	80.7			–			7.1	56.4 ± 3.8	25.8 ± 0.64
*Mean*	80 ± 1.5						7.5 ± 0.5	59 ± 10	25.4 ± 5.3

a sd was 0.02–1.5 for concentrates and 0.02–0.2 for isolates.

b – means below LOD.

#### 3.1.2 Saponin and fiber content

The content of saponin, soluble and insoluble fiber varied distinctly among the concentrates. The difference in saponin content and soluble fiber are considerably larger with CVs of 30.8 % and 28.6 %, respectively, while the CV for insoluble fiber is 10.6 %. The fiber content of different PP ingredients is rarely reported and, therefore, the fiber impact on protein functionality is largely unknown. Pelgrom et al. (2015) investigated the functional properties of different mildly refined PP fractions with various fiber content including a commercial PP isolate with 72.1 % protein and 21.5 % fiber content. The mean value of the insoluble fiber content for 12 PP concentrates (mean protein content of 55.2 %) was 13.08 % and 16.2 % for 30 PP isolates (mean protein content of 78.1 %), while the soluble fiber content was below 0.1 % for both ingredient types (Bhuiyan et al., 2024). As seen in Table 1, our results on the content of insoluble fiber in the PP concentrates are on par with the mean value for 12 different PP concentrates. Karacter had the lowest content of soluble fiber and a high content of insoluble fiber, while Kaplan had the highest content of soluble fiber and low content of insoluble fiber (Table 1). However, no direct relationship between the content of soluble and insoluble fibers are observed, since other cultivars had both high content of soluble and insoluble fibers, e.g. Akooma and Manager.

Saponins are surface active compounds with foaming properties but are also associated with bitterness (Güçlü-Üstündağ and Mazza, 2007). Peas exhibit variation in their saponin composition, but saponin B and DDMP saponin represent the most abundant and well-characterized saponins (Reim and Rohn, 2015). Moreover, differences in the content of saponin B and DDMP saponin in six pea varieties were found with a predominant content in the hulls compared to the peeled peas (Reim and Rohn, 2015). The total saponin content in peas was considerably higher (0.1–1 mg/g d.m.) (Reim and Rohn, 2015) than the content in the PP concentrates (Table 1). As seen (Table 1), Greenway had an extremely low content of saponins compared to the other cultivars, which were above 100 mg/kg (except Akooma), and with Kaplan having an extremely high saponin content. The PP isolates did not contain measurable amounts of saponins, likely not being extractable by alkaline extraction process. Reim and Rohn (2015) found that the DDMP saponin was not stable and factors like temperature, extraction solvent, and pH value, would convert DDMP saponin into saponin B and maltol.

#### 3.1.3 pH of PP ingredients

For protein ingredients obtained by the dry fractionation process, chemicals are not used, and the pH of the protein concentrate corresponds to the neutral pH of the raw material. In wet fractionation process, protein extraction is based on protein solubilization and precipitation via pH changes, consequently changing the final pH. The applicability of PP ingredients in foods depends on the food's pH: acidic pH like soft drinks, medium pH like bread, dairy, and meat products, or neutral to slightly alkaline pH such as egg-based foods. The functionality of proteins is strongly dependent on their pH, hence the neutral pH should be reported, as it can significantly differ even between batches of the same commercial PP product. Unfortunately, in many relevant studies, the neutral pH has not been reported. However, some authors have reported the pH value of commercial PP isolates. Othmeni et al. (2024) found that commercial PP isolate had a neutral pH of 7.5 (5 % w/v in protein), while a range of 6.60–7.56 for nine commercial PP isolates was reported by (Jakobson et al., 2023). A wider pH range of 5.80–7.72 (3 % wt% dispersion) was measured for five different commercial PP isolates (Ebert et al., 2020). The higher pH value for the PP isolates may be ascribed to the residual salts remaining after isoelectric extraction, where protein is precipitated by the addition of acid, separated from the rest of the solution, and the precipitated protein is re-solubilized by adjusting to pH 7.

#### 3.1.4 Particle size distribution

The particle size distributions of the PP ingredients showed different ranges with the isolates covering a broader particle range, ∼ 2–300 μm, compared to the concentrates with a narrower range of ∼ 2–120 μm, resulting in major differences between the corresponding D[4,3] and D[3,2] values, see Table 1. In general, the D[4,3] and D[3,2] for the PP isolates are markedly higher than for the PP concentrates due to the presence of larger particles (aggregates) in the isolates. However, the same cultivars did not result in large particles after processing into concentrates and isolates: Akooma and Ingrid concentrates contained markedly larger particles than the other concentrates, while the Greenway and Manager isolates contained very large particles compared to the others. Based on the DSC results, the concentrates consist of native starch granules and protein bodies as well as different sizes of cellular material and protein-cellular compounds attachments (Pelgrom et al., 2013). During the wet extraction and following drying, proteins are prone to denaturation and aggregation to different extents, seen as a greater degree of variation in particle sizes (both D[4,3] and D[3,2]) between cultivars for the protein isolates. A similar variation in the particle size of different commercial PP ingredients was reported by Sun et al. (2023) with D[4,3] values ranging from 6.2 to 42.0 μm. The large size differences between D[4,3] and D[3,2] for protein isolates are common for samples with a broad or multimodal distribution.

#### 3.1.5 Protein composition

PPs can be classified into four major fractions: globulin (soluble in diluted salt solution), albumin (soluble in water), prolamin (soluble in alcohol), and glutelin (insoluble) according to the Osborne classification (Osborne, 1907). The globulins are the main storage proteins, accounting for 55 %–65 % of the total protein, which can be further subdivided into the two main types, 18 %–52 % legumin (11S) and 31 %–73 % vicilin (7S), and a small amount (0 %–12 %) of convicilin (Boye et al., 2010). The electrophoretic analysis established that the molecular weight of the detected protein bands ranged between 3.5 and 96 kDa in agreement with the literature (Shevkani et al., 2015; Sun et al., 2023). For all 10 cultivars, the protein profiles were mainly dominated by the 11S and 7S globulins in both the PP concentrates and isolates. In addition, the concentrates were characterized by fewer and weaker protein bands, mainly migrating around 24 and < 10 kDa, and assigned to albumin 2 and 1, respectively (Croy et al., 1984; Grossmann, 2023). It was observed that large aggregates not able to permeate the gel stayed at the top of the PP isolate gels and are attributed to denatured proteins as established by DSC.

**Fig. 2:**
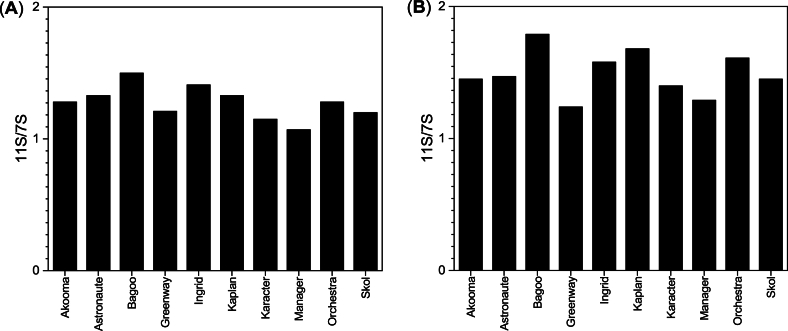
The relative ratio of 11S/7S based on SDS-PAGE analysis. (**A**) PP concentrate and (**B**) PP isolate.

The ratio of legumin to vicilin (11S/7S) has been reported to have a wide variability, ranging between 0.1 and 5, depending on various factors such as cultivars, cultivation, environmental conditions, and protein extraction method (Barac et al., 2010; Choi et al., 2022; Husband et al., 2024). The dependency of the globulin protein type on solubility is unclear. Kimura et al. (2008) and Husband et al. (2024) found that 7S was more soluble than 11S at pH below 7, whereas Liang and Tang (2013) found that 11S exhibited higher solubility than 7S at pH below 5. The difference in these findings may be due to differences in the extraction methods used for protein isolation. Green pea 11S was reported to be more soluble than 7S at pH below 5, but changed at pH above 6, where 7S became more soluble than 11S (Chang et al., 2022). Interestingly, the green pea vicilin fraction had markedly higher foaming capacity compared to the legumin fraction at acidic (pH 3), neutral (pH 7), and basic (pH 9) conditions, while the foam based on the legumin fraction was more stable at all pHs (Chang et al., 2022). As observed in Fig. 2, nearly all of the 10 investigated cultivars have a higher amount of 11S than 7S in both types of ingredients. Moreover, the 11S/7S ratio varies only slightly among cultivars and ingredient type (concentrates vs isolates), though few cultivars have a marginally higher content of 11S in their isolate.

**Fig. 3:**
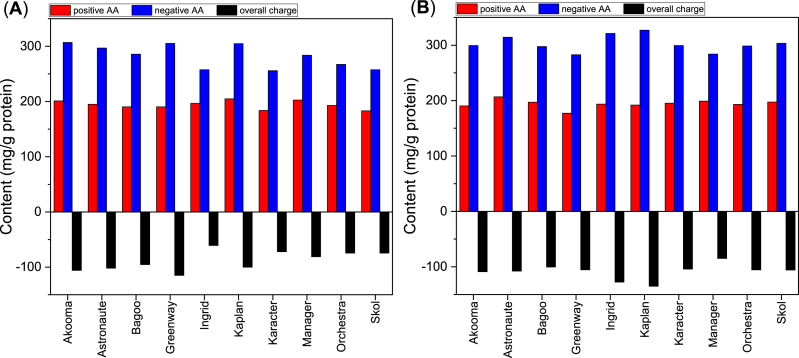
The content of positive (R + H + K) and negative (D + E) amino acids is based on the AA composition results as shown in Table 2. The overall charge is the arithmetic sum of the content of the positive and negative amino acids. (**A**) PP concentrate and (**B**) PP isolate.

The amino acid profiles showed inherent variations among cultivars in both PP concentrates and isolates, see Table 2. The amino acid compositions vary considerably and may influence the physico-chemical and functional properties of the PP ingredients due to the variation in the content of the positive amino acids and the negative amino acids resulting in different total charge as shown in Fig. 3. For example, Tang et al. (2021) concluded that isolates (extracted in the laboratory) of several common pulses, including pea, had higher solubility due to a higher content of the charged amino acids lysine, histidine, aspartic acid, and glutamic acid.

**Table 2:** Relative amino acid composition (%) of the PP ingredients.

AA^a^	A	R	D	Cx	E	G	H	I	L	K	Mx	F	P	S	T	W	Y	V
**Cultivar**																		
***Concentrate***																		
Akooma	4.4	9.7	11.6	1.0	19.3	4.4	2.7	4.7	5.7	7.8	0.9	5.1	4.2	5.0	4.1	0.7	3.7	5.2
Astronaute	4.1	9.8	12.5	1.0	18.1	4.6	2.7	4.6	5.8	7.6	0.9	5.3	4.3	5.1	3.8	0.7	3.9	5.2
Bagoo	4.5	9.2	11.4	1.0	18.4	4.5	2.7	5.0	6.0	8.0	0.9	5.3	4.1	5.1	3.9	0.7	4.0	5.3
Greenway	4.4	8.6	12.7	0.9	18.1	4.8	2.6	5.1	6.1	8.1	0.8	5.5	4.0	5.0	4.1	0.6	3.6	5.1
Ingrid	4.5	9.7	10.2	1.1	16.9	4.7	3.0	4.8	8.1	8.0	1.0	5.4	4.4	4.7	3.8	0.7	3.8	5.2
Kaplan	4.1	11.6	13.1	0.8	18.2	3.8	2.2	4.4	7.6	7.2	0.8	5.0	4.0	4.7	3.3	0.6	3.4	5.1
Karacter	4.5	10.6	11.6	1.0	17.0	3.8	2.5	4.8	7.9	7.5	0.9	5.1	4.3	5.3	3.7	0.7	3.7	5.0
Manager	4.5	9.7	11.6	1.1	17.2	3.7	2.8	4.8	7.9	8.1	1.0	5.2	4.2	5.2	3.5	0.7	3.7	5.2
Orchestra	4.3	10.3	11.6	1.1	17.0	3.7	2.6	4.7	7.8	7.7	0.9	5.4	4.2	5.0	3.9	0.7	3.9	5.2
Skol	4.4	9.8	11.3	1.2	16.9	4.2	2.4	4.7	8.1	7.9	1.0	5.4	4.4	5.2	3.5	0.7	3.8	5.0
*Mean*	4.4 ± 0.1	9.9 ± 0.8	11.8 ± 0.8	1.0 ± 0.1	17.7 ± 0.8	4.2 ± 0.4	2.6 ± 0.2	4.8 ± 0.2	7.1 ± 1.1	7.8 ± 0.3	0.9 ± 0.1	5.3 ± 0.2	4.2 ± 0.1	5.0 ± 0.2	3.8 ± 0.2	0.7 ± 0.0	3.7 ± 0.2	5.1 ± 0.1
***Isolate***																		
Akooma	4.4	9.7	12.3	0.9	18.2	4.0	2.6	5.0	8.0	7.1	1.0	5.3	4.1	4.7	3.2	0.7	3.7	5.1
Astronaute	3.9	9.5	12.3	0.9	17.6	4.0	2.5	5.0	8.1	7.6	0.9	5.4	4.3	5.0	3.6	0.6	3.8	5.1
Bagoo	4.3	9.5	11.5	0.9	18.2	3.7	2.5	5.0	8.2	7.7	1.0	5.5	4.0	5.1	3.5	0.7	3.6	5.2
Greenway	4.3	8.4	13.0	0.9	16.4	3.6	2.4	5.3	8.7	7.7	1.0	5.8	4.3	5.0	3.5	0.6	3.8	5.3
Ingrid	4.3	9.0	13.0	0.9	18.2	3.5	2.5	5.0	7.9	7.3	1.0	5.3	4.0	5.4	3.3	0.7	3.7	5.1
Kaplan	3.9	9.0	12.5	0.8	19.1	4.0	2.5	4.9	8.3	7.1	0.8	5.3	4.1	5.2	3.2	0.6	3.7	5.1
Karacter	4.2	9.1	10.9	0.9	18.4	3.6	2.5	5.1	8.2	7.5	1.0	5.5	4.5	5.2	3.4	0.6	3.8	5.4
Manager	4.1	9.5	11.8	0.8	16.4	4.1	2.5	5.2	8.4	7.7	1.0	5.6	4.3	4.9	3.4	0.7	3.8	5.7
Orchestra	3.9	9.4	12.2	0.9	17.9	4.1	2.5	5.1	8.3	7.6	0.9	5.4	4.1	4.9	3.1	0.6	3.7	5.2
Skol	4.0	9.5	12.2	1.0	17.5	4.0	2.4	5.0	8.2	7.4	1.0	5.5	4.1	4.8	3.6	0.6	3.8	5.3
*Mean*	4.1 ± 0.2	9.3 ± 0.4	12.2 ± 0.6	0.9 ± 0.1	17.8 ± 0.9	3.9 ± 0.2	2.5 ± 0.1	5.1 ± 0.1	8.2 ± 0.2	7.5 ± 0.2	1.0 ± 0.1	5.5 ± 0.2	4.2 ± 0.2	5.0 ± 0.2	3.4 ± 0.2	0.6 ± 0.0	3.7 ± 0.1	5.2 ± 0.2

a One letter code; A: Alanine, R: Arginine, D: Aspartic acid, Cx: Cysteic acid, E: Glutamic acid, G: Glycine, H: Histidine, I: Isoleucine, L: Leucine, K: Lysine, Mx: Methionine sulfone, F: Phenylalanine, P: Proline, S: Serine, T: Threonine, W: Tryptophan, Y: Tyrosine, V: Valine ^2^ Hydrophilic amino acid.

#### 3.1.6 Zeta potential

**Fig. 4:**
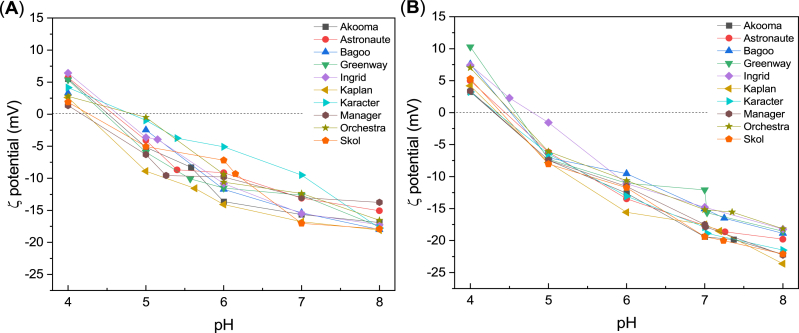
ζ-potential (mean values) of PP ingredients as a function of pH. (**A**) PP concentrate and (**B**) PP isolate. Standard deviations are in the range 0.26–1.1 mV. Broken line at y = 0 is to guide the eye regarding the isoelectric point (Table 3).

The ζ-potential of a protein may be more appropriate in relation to the protein's techno-functionality, since this parameter describes the overall surface charge, thus the capability of electrostatic interaction with other molecules. The ζ-potential is a result of different intrinsic factors, including the amino acid content and conformation of proteins, and the extrinsic solvent conditions like pH, ionic strength, and temperature. As observed from Fig. 4 The change in pH has a large effect on the ζ-potential of the PP concentrates and isolates. The ζ-potential of the concentrates differs considerably within cultivars at pH between 5 and 7, while the ζ-potential of the isolates is more similar. At pH 7.0, the concentrates had ζ-potentials ranging between −9.50 and −17.0 mV and the isolates had ζ-potentials ranging between −11.1 and −19.4 mV (Fig. 4). It has been concluded that a generic difference is present between pea albumins and globulins at pH 7.0, as the albumins had ζ-potentials ranging from −3.3 to −2.0 mV, while globulins were more negative with ζ-potential ranging from −14.8 to −10.3 mV (Yang et al., 2022). Moreover, it has been previously reported that vicilin (extracted in the laboratory) from pea has a lower net ζ-potential (−19.7 mV) compared to legumin (−26.7 mV) (Sha and Xiong, 2022). Salt extraction (at laboratory scale) followed by dialysis also resulted in a pea fraction with a majority of vicilin and possessed the lowest net ζ-potential (−16.2 mV) (Yang et al., 2021). Stone et al. (2015a) found that the surface charge of the isolate (extracted in the laboratory) from seven different pea cultivars ranged from −23.1 to −26.8 mV at pH 7.0. In addition, all isolates, regardless of cultivar, had similar isoelectric points at pH between 4.6 and 4.9 (Stone et al., 2015a). The ζ-potentials of the concentrates and isolates from the ten cultivars in this study were comparable to previously reported values; however, the isolates are slightly more negative than the concentrates at higher pH (Fig. 4). The overall charge of the amino acid composition is correlated with the ζ-potential at natural pH for the PP concentrates, but not for the PP isolates (Figs. S1 and S2). The ζ-potential of the concentrates and isolates likely differ due to the structural differences, with concentrates containing native proteins, while the isolates contain denatured and aggregated protein particles. The distribution of charged groups is distinctly different between the concentrates and isolates in the pH interval 6–8, which are of most interest in food applications. The pH effect on amino acid ionization, specifically the higher capacity of protonation of carboxyl groups and de-protonation of amino groups near the protein's surface at these pHs is suggested to be due to the structural differences of the protein in the two types of PP ingredients. Furthermore, the lower net charge of concentrates may be due to the content of carbohydrates potentially neutralizing charges on the protein surface (Keivaninahr et al., 2021). Nevertheless, as observed from Table 3 the isoelectric point (pI) shows a high degree of similarity among the PP ingredients and are comparable to the pI generally found for legume proteins (pI between 4 and 5) (Boye et al., 2010).

**Table 3:** The isoelectric point of PP ingredients taken from Fig. 4.

pI	Akooma	Astronaute	Bagoo	Greenway	Ingrid	Kaplan		Karacter	Manager	Orchestra	Skol
Concentrate	4.5	4.6	4.7	4.5	4.7	4.2		4.8	4.2	4.9	4.3
Isolate	4.3	4.5	4.5	4.6	4.8	4.4		4.3	4.3	4.6	4.4

#### 3.1.7 Water and oil holding capacity

Water or oil holding properties of proteins can be important for application in meat analogs, bakery products, plant-based mayonnaise, or dairy analogs in relation to maintenance of the product quality like textural attributes and the consumer acceptability like mouthfeel and taste. The affinity of proteins for either the water or oil phase depends on the proportion of hydrophilic and hydrophobic areas on the protein surface, hence it is related to the protein's structure, and will contribute to WHC and OHC, respectively. Overall, the isolates had a considerably higher WHC than the concentrates, see Table 4. This result agrees with the finding by Keivaninahr et al. (2021) that PP isolate had higher WHC compared to concentrates. The authors suggested that protein isolates exhibit higher WHC due to a higher protein content and the presence of small starch fragments. Indeed, all PP isolates contained considerably more protein than the concentrates (Table 1). On the other hand, the concentrates contain smaller particles rendering an increase in the contact area between water and the protein particles. Consequently, there is potential for more binding sites to water. In this study, only minor differences in WHC between the cultivars within each ingredient type were found. Stone et al. (2015a) also found no differences between the WHC of isolates (laboratory extraction) from seven different pea cultivars. However, it was also found that the effect of cultivar on WHC was dependent on the extraction method for obtaining the isolates (Stone et al., 2015b). It has been suggested that a higher WHC arises from the increased hydrogen bonding with water by the polar and ionic protein side chain groups being exposed at the protein surface due to the specific extraction technique (Adebowale et al., 2011; Stone et al., 2015b). However, no consistent trends could be observed for the WHC results and relation with protein content and net charge. There was no direct relationship between WHC and protein content or ζ-potential or amino acid overall charge (Figs. S1 and S2). The higher WHC makes the PP isolate a more suitable ingredient for products where hydration is needed, such as bread, cakes, and muffins. The Orchestra and Skol isolates have the highest WHC suggesting possible application in bakery products and meat analogs.

**Table 4:** Water and oil holding capacities of the PP ingredients.

Cultivar	WHC (g water/g powder)	OHC (g oil/g powder)
***Concentrate***		
Akooma	1.93 ± 0.052	1.65 ± 0.075
Astronaute	2.39 ± 0.063	1.47 ± 0.042
Bagoo	2.00 ± 0.093	1.73 ± 0.034
Greenway	2.18 ± 0.077	1.66 ± 0.022
Ingrid	2.23 ± 0.22	1.67 ± 0.082
Kaplan	2.12 ± 0.10	1.59 ± 0.098
Karacter	1.95 ± 0.039	1.67 ± 0.11
Manager	2.53 ± 0.18	1.61 ± 0.041
Orchestra	2.11 ± 0.18	1.43 ± 0.045
Skol	2.16 ± 0.13	1.78 ± 0.008
*Mean*	2.16 ± 0.19	1.63 ± 0.11
***Isolate***		
Akooma	6.80 ± 0.37	3.79 ± 0.018
Astronaute	7.33 ± 0.34	2.91 ± 0.083
Bagoo	7.09 ± 0.006	3.29 ± 0.020
Greenway	7.35 ± 0.15	3.43 ± 0.12
Ingrid	7.64 ± 0.020	2.99 ± 0.2
Kaplan	7.85 ± 0.25	4.23 ± 0.061
Karacter	7.73 ± 0.48	4.01 ± 0.063
Manager	7.69 ± 0.15	3.66 ± 0.12
Orchestra	7.99 ± 0.10	3.66 ± 0.008
Skol	7.99 ± 0.013	3.39 ± 0.042
*Mean*	7.54 ± 0.40	3.54 ± 0.42

The OHC was lower than the WHC, see Table 4. Similar to WHC, the OHC was dependent on the ingredient type with higher values for PP isolates than PP concentrates, but not dependent on the cultivar. In the same context, Stone et al. (2015a) did not find a difference in the OHC of isolates from seven different pea cultivars, whereas the method of laboratory production of these isolates did affect their OHC (Stone et al., 2015b). The capacity of a protein powder to absorb oil depends on factors such as hydrophobicity for binding to lipid molecules and the pore structure for physical entrapment of oil (Keivaninahr et al., 2021; Stone et al., 2015a). In products where proteins are the main linkages between lipids and water, like sausages, cold cuts, and sauces, the PP isolates with high WHC and OHC will be a more suitable ingredient compared to PP concentrate. In these applications the isolates from Kaplan and Karacter could be useful due to their additional high OHC.

#### 3.1.8 Sensory evaluation of PP concentrates

**Fig. 5:**
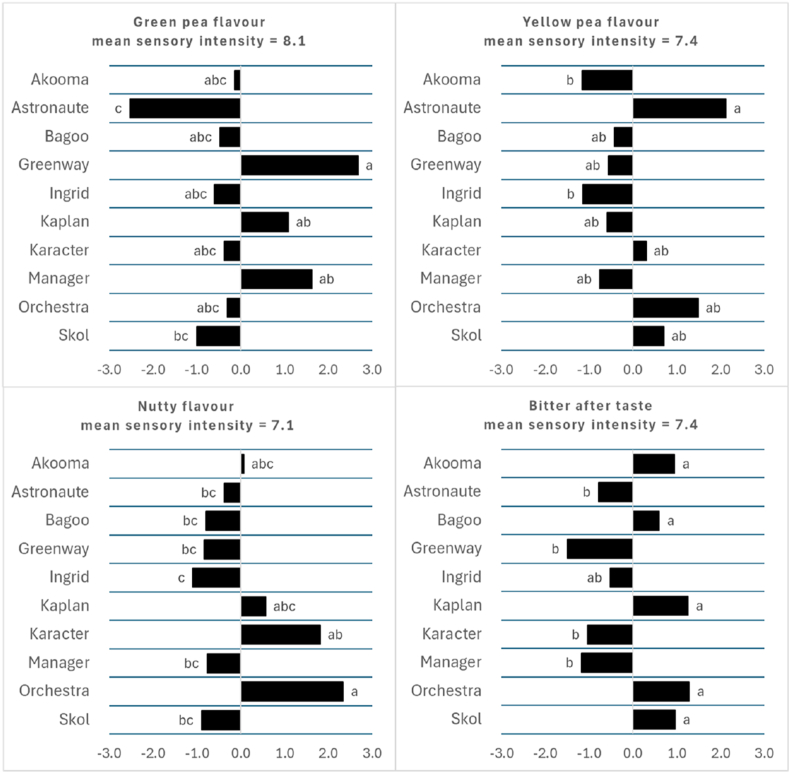
The sensory attributes of the ten PP concentrates showing significant (p < 0.05) differences between the cultivars. The bar diagrams represent the sensory intensity differences from the panel mean. Different letters show significant differences between the respective cultivars.

The sensory evaluation provided important insights into desirable and less desirable sensory properties of the raw PP ingredients, which may improve or cause rejection of PP end-products. The PP concentrates were generally perceived as ‘sweet’ (mean intensity of 9.3 (SD = 0.8)) with lower intensities for ‘umami’ (mean value = 7.7 (SD = 0.7)) and ‘bitter’ taste (mean intensity = 6.5 (SD = 0.5)). ‘Astringency’ was also clearly noticeable with a mean intensity of 7.7 (SD = 0.8). The sensory evaluation showed only 4 attributes differing between the PP concentrates from the different cultivars (Fig. 5). These were ‘green pea flavor’ (p < 0.001), ‘yellow pea’ flavor (p = 0.003), ‘nutty’ flavor (p = 0.007), and ‘bitter’ after taste (p = 0.035). The Astronaute concentrate was characterized by the highest ‘yellow pea’ and lowest ‘green pea’ flavor, whereas the Greenway cultivar gave a more marked ‘green pea’ flavor compared to the Skol and Astronaute cultivars. The Orchestra and Karacter had more ‘nutty’ flavor character compared to most other cultivars (Fig. 5). The PP concentrates tasted generally ‘bitter’, which was most noticeable in the aftertaste. Greenway, Manager, Karacter, Astronaute concentrates were rated significantly lower in ‘bitter’ aftertaste, with Ingrid showing no differences, compared to the other cultivars. The PP concentrate from the Ingrid cultivar tended to have the least intense flavors. No clear correlation between the content of soy saponins and the bitter after taste could be observed (R2 = 0.136). Saponin B and the mixture (20:80) of Saponin B and DDMP have shown to taste bitter at 4 mg/L with the bitter taste of the mixture most markedly rising with increasing concentration. They have been found at relatively high levels (1.1–2.5 g/kg) in dry peas (Heng et al., 2006). However, the levels found in the PP concentrate in the present study were about a factor 10 lower and since the saponins did not correlate with bitter after taste, other components seem to be more important to cause the bitter after taste.

The three key sensory attributes, ‘bitter’, ‘beany’, and ‘roasted’, were special evaluated for heat-treated PP concentrates to test the sensory promise in heat-treated end-products. Some cultivars showed clear changes on heat treatment of the PP concentrates (Table 5). ‘Bitter’ taste significantly increased (p < 0.05) for Astronaute (61 %), while it significantly decreased (−30 %) to a moderate level for Manager with a similar tendency for Kaplan (−24 %). ‘Beany’ flavour was not affected by the heat treatment, whereas ‘roasted’ flavour significantly increased for Astronaute (51 %) and Bagoo (64 %) PP concentrates with a similar tendency for Kaplan (50 %). The changes in ‘bitter’ taste upon thermal treatment were not correlated (R2 = 0.18) with the saponin content (Table 1), even though saponins in acidic environment are thermally unstable due to hydrolytic cleavage of the glycosidic groups (Reim and Rohn, 2015). The changes in roasted flavour may be caused by Maillard-type and protein-degrading reactions. The PP concentrate of the Astronaute cultivar was most affected by the heat treatment.

**Table 5:** Relative sensory intensity changes (%ΔI) in key attributes between raw and heat-treated PP concentrates (ΔT of 165 °C for 5 min).

Cultivar	Bitter taste		Beany flavour		Roasted flavour	
	ΔI	p-value	ΔI	p-value	ΔI	p-value
Akooma	16 %	0.479	1 %	0.956	3 %	0.919
Astronaute	61 %	**0.034**	8 %	0.285	51 %	**0.010**
Bagoo	−12 %	0.542	10 %	0.974	64 %	**0.003**
Greenway	8 %	0.709	1 %	0.962	23 %	0.374
Ingrid	−24 %	0.184	21 %	0.250	18 %	0.471
Kaplan	−24 %	0.057	12 %	0.414	50 %	0.075
Karacter	−26 %	0.229	8 %	0.620	15 %	0.516
Manager	−30 %	**0.024**	−15 %	0.210	30 %	0.219
Orchestra	−3 %	0.904	−6 %	0.519	20 %	0.266
Skol	17 %	0.575	−17 %	0.106	−5 %	0.768

#### 3.1.9 Protein solubility

**Fig. 6:**
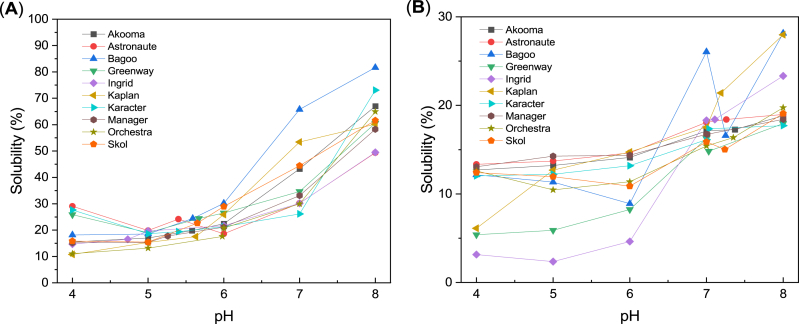
Protein solubility (mean values) of PP ingredients as a function of pH. Standard deviations are in the range 0.08–2.47 %. (**A**) PP concentrate and (**B**) PP isolate. Notice the difference in solubility scales.

Protein solubility is important for protein beverages, such as plant-based milk substitutes. Moreover, it is not clarified if protein solubility is a prerequisite for protein functional properties like foamability and emulsification. The pH-dependent (at food relevant pH from 4 to 8) protein solubility profiles of the PP concentrates and isolates are presented in Fig. 6. A typical pH dependency for plant storage proteins with minimal solubility values around pH 4 and clearly higher solubility upon reaching pH 8 is observed for both types of ingredients. At pH above 4, the protein solubility of the concentrates is considerably higher than the protein solubility of the isolates (Fig. 6). This finding corroborates with other results that commercial pea concentrates exhibited higher solubility than commercial pea isolates (Keivaninahr et al., 2021; De Angelis et al., 2024). In addition, different solubility trends are observed within the cultivars over the whole pH range for both ingredient types. Similar trends for pea proteins have been reported before. Barac et al. (2010) found different solubility of six pea cultivar isolates, though a higher solubility at pH 7 and 8 compared to solubility at pH 3 and 5 were obtained for all cultivars. A cultivar effect on the solubility of 7 different isolates (Stone et al., 2015a) and an additional effect of the extraction method (Stone et al., 2015b) were also found. Protein solubility in an aqueous medium is dependent on physico-chemical characteristics and thermodynamic interactions with water, such as content of hydrophilic amino acids, surface charge, and particle size. A higher solubility of different PP isolates was attributed to a higher relative content of hydrophilic amino acids (Sun et al., 2023; Tang et al., 2021). However, the protein solubility at the natural pH of the PP concentrates and isolates (Fig. 6) did not correlate with the content of hydrophilic amino acids. Proteins with a high surface charge will experience electrostatic repulsion and, thereby, exhibit better solubility. As seen in Fig. 6, both concentrates and isolates exhibit lowest solubility at pH around the pI (Table 3), where the proteins are zero charged and experience less repulsive interactions. This may in turn result in increased protein-protein interaction causing agglomeration and reduced solubility. The pea proteins’ net charge increases upon increasing pH (Fig. 2) resulting in stronger electrostatic repulsion and enhancing solubility. Yet, the lack of high correlation between solubility and ζ-potential at the respective pHs (Appendix A, Figs. S1 and S2) underlines the importance of other physico-chemical characteristics on protein solubility. As mentioned previously, the different ingredient processing methods, dry fractionation versus wet extraction, resulted in different particle size distributions of the powders (Table 1). Especially, the wet extraction produced larger particles with reduced specific surface area compared to the smaller particles in the concentrates. A larger surface area may decrease the contact area between water and proteins, thereby decreasing the sites for protein-water interactions, resulting in the lower protein solubility observed for the isolates. Solubility was found to be negatively correlated with particle size of a commercial PP isolate under neutral and acidic pH (Othmeni et al., 2024). The solubility at neutral pH of the concentrates and isolates did, however, not correlate with their particle sizes (Figs. S1 and S2). The considerably higher protein solubility of the PP concentrates offers new possibilities for plant-based beverage application at neutral pH. Notably, the concentrates produced from the cultivars Bagoo, Kaplan, Skol, and Akooma had superior solubility at pH 7.

### 3.2 Foamability

Foam formation and stability are important requirements to produce food products where aeration is required, like ice cream, whipped toppings, cakes, and meringues, therefore the foamability of the PP ingredients is essential to their application in the production of such non-animal product types.

#### 3.2.1 Foam capacity

**Fig. 7:**
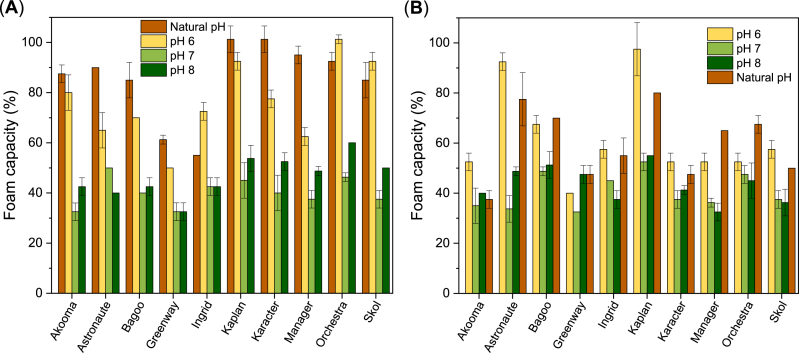
Foam capacity (mean values) of PP ingredients as a function of pH (natural pH see Table 1). Standard deviations represented by error bars. (**A**) 3 % (w/v) PP concentrate and (**B**) 1.5 % (w/v) PP isolate.

Fig. 7 shows that ingredients prepared from all cultivars are characterized by notably different FC, measured as foam overrun, ranging between 30 % and 100 %. However, overall, the FC of the concentrates ranged higher between 40 and 70 % than isolates with FC between 30 and 60 %, demonstrating a slightly better ability of the concentrates to form foams. The FC of the PP ingredients was highly pH-dependent for both PP concentrates and isolates. In general, FC increased in the following order: pH 7 < pH 8 ≪ pH 6 < natural pH for both types of ingredients (Fig. 7). For some ingredients the FC at pH 6 was slightly higher than FC at natural pH. It is emphasized that natural pH range for concentrates is 5–6, while for isolates the natural pH range is 7–8 (Table 1). In the case of the concentrates, the two cultivars Kaplan and Karacter were superior foaming ingredients, whereas Greenway and Ingrid were less effective in forming foam at their natural pH resulting in the lowest FC compared to FC of the other concentrates at their natural pH. For the isolates, the two cultivars Astronaute and Kaplan, on the other hand, exhibited noticeably higher FC compared to the other cultivars.

One of the differences between the concentrates and the isolates not related to the protein as such is the content of saponins. The content of saponins in the concentrates may have a positive effect on FC compared to the isolates which do not contain saponins. A slight correlation (correlation coefficient 0.62) between saponin content and FC was also found (Appendix A, Fig. S1). However, Góral et al. (2021) found that despite a large difference in the saponin content of different soybean extracts they exhibited similar surface activity. Therefore, the different FC values may primarily be attributed to the PPs' properties. During the foaming process, the PPs must be able to adsorb onto the air-water interface to form a cohesive, flexible, and continuous film surrounding air bubbles via protein-protein interaction. Hence, the foam formation is determined by the characteristics of this interfacial film, which is related to the proteins’ surface activity, structure, and size, yet the question of which property may best relate to foaming functionality remains unanswered. Other studies corroborate the cultivar difference in FC, albeit these investigations are only on lab-produced PP isolates (Barac et al., 2010; Stone et al., 2015b; Cui et al., 2020). The FC has been found to be dependent on the protein concentration of the system (Aluko et al., 2009; Chao and Aluko, 2018). However, it is emphasized that in our study the FC is based on 3 % concentrate and 1.5 % isolate solutions resulting in comparable PP concentrations in the systems. The effect of pH on FC has been reported for both lab-extracted and commercial PP isolates, where the lowest FC was observed at pH 5 in all studies (Barac et al., 2010; Chao and Aluko, 2018; Othmeni et al., 2024). Chao and Aluko (2018) and Othmeni et al. (2024) attributed the low foamability to protein aggregation near the pI, thus negatively affecting the PP ability to unfold and entrap air bubbles. The presence of insoluble protein aggregates in the isolates may interfere with the foam formation mechanisms. The higher foaming ability of concentrates in comparison to isolates was explained by a higher content of highly soluble albumin-type proteins, which are better preserved during dry fractionation compared to wet extraction, where the albumin fraction is separated from the globulin fraction (De Angelis et al., 2024). However, conclusions on the role of protein solubility on foam capacity are not clear. Stone et al. (2015b) thus reported a positive correlation between protein solubility and FC, indicating that when more soluble proteins can migrate to the air-water interface, more foam is formed. In contrast, Barac et al. (2010) and Cui et al. (2020) found no correlation between foaming properties and solubility of PP isolates. In agreement with the latter result, no correlation was found between protein solubility and FC for neither PP concentrates or isolates (Appendix A, Figs. S1 and S2). Nonetheless, the proteins in the PP concentrates possessed a structural conformation at higher pH suitable for unfolding and enhanced flexibility during the foaming process, hence better interfacial film formation, possible due to their native state.

#### 3.2.2 Foam stability

**Fig. 8:**
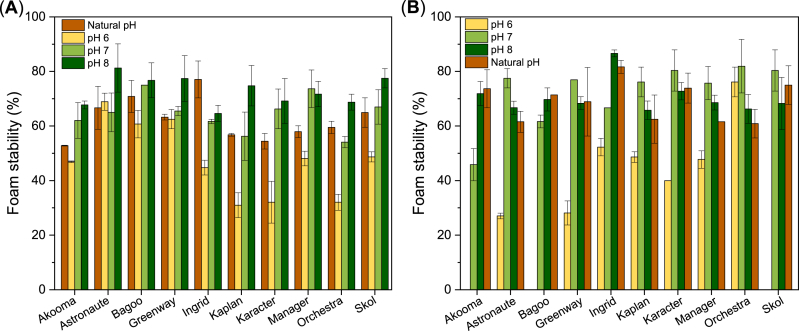
Foam stability (mean values, measured as foam volume after 30 min) of PP ingredients as a function of pH (natural pH see Table 1). Standard deviations represented by error bars. (**A**) 3 % (w/v) PP concentrate and (**B**) 1.5 % (w/v) PP isolate.

Foam instability and collapse occur due to gravitational liquid drainage, bubble coalescence, and/or disproportionation. The foam stability (FS) is, thus, related to the continuous phase and properties of the interfacial film around the bubble, and adhesive, elastic, and airtight membranes results in improved FS. Fig. 8 shows that FS of the concentrates was less dependent on pH in comparison to the FC of the concentrates. The isolates also exhibit a degree of independence on FS at pH 7, 8, and natural pH, whereas FS is highly influenced by pH 6. The foam of Akooma, Bagoo, and Skol were highly unstable and collapsed within 30 min of measurement at pH 6. A high foam stability has been attributed to protein concentration (Barac et al., 2010; Chao and Aluko, 2018; Cui et al., 2020), protein composition (Chang et al., 2022; De Angelis et al., 2024), and protein charge (Chao and Aluko, 2018; Othmeni et al., 2024). A high protein concentration could result in more polypeptide chains being available to form the interfacial film, thereby leading to increased strength and stability. The side-by-side packed α-helix structure of 11S may form a thick and cohesive interfacial film that prevents bubble instability. The net charge of proteins affects interactions between air bubbles in the foam; hence higher charge of the interfacial film reduces interactions between the air bubbles resulting in stable bubble distribution and thereby foam. However, the FS of the cultivars at different pHs did not show correlation with either protein content, protein solubility, ζ-potential, nor L/V ratio (Appendix A, Figs. S1 and S2). Other possible explanations can be ascribed to multiple factors related to foam methods, the protein conformation, and the presence of non-protein compounds. A high protein surface hydrophobicity can result in increased aggregation potentially disrupting foam structure and reducing stability (Othmeni et al., 2024). In addition, the carbohydrates in the concentrate may enhance the viscosity of the continuous phase, thereby being bulky barriers between the air bubbles, and preventing both liquid drainage and bubble coalescence and disproportionation, eventually inhibiting foam destabilization (De Angelis et al., 2024).

### 3.3 Principal component analysis

**Fig. 9:**
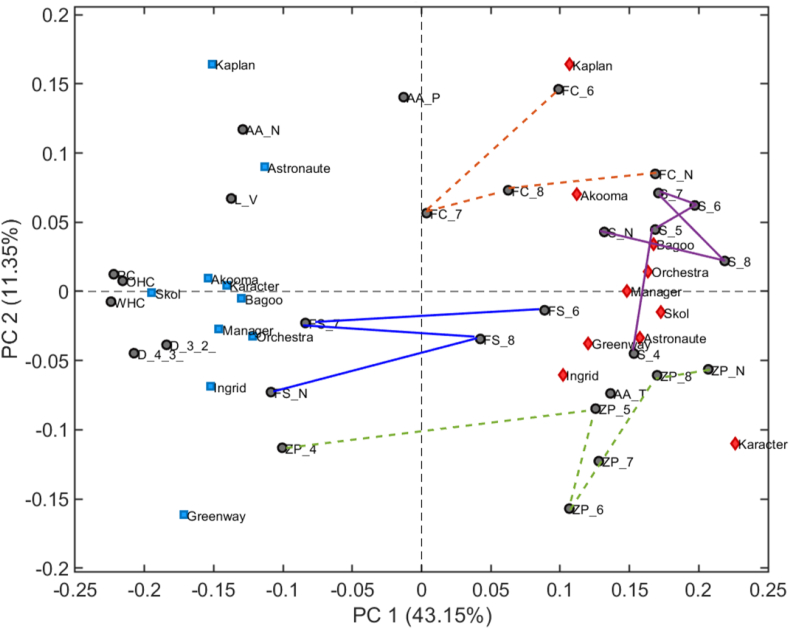
Principal component analysis (PCA) biplot (autoscaled) of the powder characteristics and foam properties of the PP concentrates (red) and isolates (blue). FC-N foam capacity at natural pH, FC-6 foam capacity at pH 6, FC-7 foam capacity at pH 7, FC-8 foam capacity at pH 8, FS-N foam stability at natural pH, and connecting lines are to guide the eye. FS-6 foam stability at pH 6, FS-7 foam stability at pH 7, FS-8 foam stability at pH 8, S-N protein solubility at natural pH, S-4 protein solubility at pH 4 (connecting lines to guide the eye). S-5 protein solubility at pH 5, S-6 protein solubility at pH 6, S-7 protein solubility at pH 7, S-8 protein solubility at pH 8 (connecting lines to guide the eye). ZP-N ζ-potential at natural pH, ZP-4 ζ-potential at pH 4, ZP-5 ζ-potential at pH 5, ZP-6 ζ-potential at pH 6, ZP-7 ζ-potential at pH 7, ZP-8 ζ-potential at pH 8 (connecting lines to guide the eye). D[3,2] surface average diameter, D[4,3] volume average diameter, PC protein content, L/V 11S/7S ratio, AA-P positive amino acids, AA-N negative amino acids, AA-T overall charge, WHC water holding capacity, OHC oil holding capacity.

Principal component analysis, shown in Fig. 9, was conducted to provide a global overview of the relationships between the powder and physico-chemical characteristics and the foamability of the 20 different PP ingredients. The first two principal components explain 54.4 % of the variability with 43.1 % PC1 and 11.3 % PC2. PP concentrates scored positively on PC1 and are located to the right of the origin, whereas PP isolates scored negatively on PC1, positioned to the left. This separation highlights distinct differences in composition, physico-chemical characteristics, and functional properties between the two types of PP ingredients. Furthermore, the PP varieties are dispersed along PC2, with cultivars scoring both positively and negatively, indicating variability within both PP concentrates and isolates. Notably, PP isolates cluster more closely along PC2, suggesting greater similarity in their measured properties compared to the more scattered PP concentrates. The PP isolates have the highest protein content (PC), the largest particle size (D[4,3] and D[3,2]), and the highest water holding capacity (WHC) and oil holding capacity (OHC). The strong intercorrelation between isolate particle sizes and water and oil holding capacities indicate that good holding capacities depend on larger protein aggregates, likely due to voids in the structure enabling ‘trapping’ of water or oil molecules. However, these parameters are not associated with foaming capacity. The concentrates are instead characterized by higher protein solubility (S) and zeta-potential (ZP, except ZP-4). Moreover, the processing method, dry fractionation versus wet extraction, seems to significantly impact on the types of the PPs accumulated in the ingredients, since the concentrates are characterized by higher overall amino acid charges (AA-T) whereas the isolates have higher total negative charges (AA-N). In addition, a larger proportion of legumins are extracted by wet extraction. The concentrates with superior solubility were positively associated with foam capacity.

## 4 Conclusions

This study demonstrated that 10 different pea cultivars exhibited clear differences in their application potential, with specific cultivars showing distinct advantages depending on the desired functionality. Kaplan produced protein ingredients with the highest protein content, while Bagoo and Manager resulted in low protein content ingredients. The processing method, dry fractionation or wet extraction, was identified as a key factor influencing protein functionality. Wet extraction led to isolates with higher protein content (78 %–83 %), pH (6.7–8.2), and larger particle sizes (43–78 μm) due to extraction-induced protein denaturation and aggregation. Whereas gentle dry fractionation led to concentrates with low protein content (41 %–54 %), pH (5.4–5.9), and smaller particles (25–32 μm), and preserved native structure of the proteins. Among the two ingredient types, pea protein concentrates consistently showed the highest solubility and foam capacity (40 %–70 %), especially at specific pH levels. Especially, the cultivars Bagoo, Skol, and Akooma displayed superior solubility making them suitable for protein dispersed application such as high-protein beverages. Kaplan and Karacter concentrates exhibited the highest foaming properties making them ideal for aerated food products, e.g. whipped toppings like barista milk. However, these concentrates were not tasteless and showed some marked differences in sensory quality. In contrast to concentrates, pea protein isolates exhibited significantly higher water (7.54 ± 0.40 g water/g powder) and oil (3.54 ± 0.42g oil/g powder) holding capacities, suggesting better performance in products needing moisture retention or fat mimicking, such as baked goods, meat alternatives, or emulsified products. Overall, this study confirms that both cultivar selection and extraction methods are crucial tools for tailoring pea protein ingredients to specific food applications, offering valuable flexibility for product developers in the plant-based sector.

## CRediT author statement

Jason D. Foley: Conceptualization, Data curation, Formal analysis, Methodology, Writing. Tomasz P. Czaja: Data curation, Formal analysis, Methodology, Writing. Maria M. Rieckmann: Data curation, Formal analysis, Methodology, Writing. Malbor Dervishi: Data curation, Formal analysis, Methodology, Writing. Søren B. Engelsen: Writing – review and editing. Kristian H. Laursen: Writing – review and editing. Andrea Bononad-Olmo: Data curation, Formal analysis. Wender L. P. Bredie: Data curation, Formal analysis, Writing. Vibeke Orlien: Conceptualization, Data curation, Formal analysis, Funding acquisition, Methodology, Project administration, Writing original draft – review and editing.

## Funding

This research was a part of the AQRIFood project, which received funding from Innovation Fund Denmark via the INNOMISSION 3 Partnership AgriFoodTure program (grant no. 1152-00001B) and 10.13039/501100009708Novo Nordisk Foundation (EcoSap, NNF20OC0060298).

## Declaration of competing interest

The authors declare that they have no known competing financial interests or personal relationships that could have appeared to influence the work reported in this paper.

## Data Availability

Data from the SDS-PAGE analysis will be made available on request.
