# Supplementary material for: Cultivar selection and processing methods are crucial tools for tailoring the physico-chemical properties and functionality of pea (Pisum sativum L.) protein ingredients
Source: Curr Res Food Sci. 2025 Oct 10;11:101218. doi: 10.1016/j.crfs.2025.101218 (PMC12553074; doi:10.1016/j.crfs.2025.101218)

**Appendix A. Supplementary data**

**Saponin LC-MS procedure**

Identification of individual saponins was performed using liquid chromatography–quadrupole time-of-flight tandem mass spectrometry (LC-qToF-MS/MS). The analysis was conducted on a Dionex UltiMate 3000 Quaternary Rapid Separation UHPLC+ focused system (Thermo Fisher Scientific, Germering, Germany) using a Kinetex 1.7 μm XB-C18 column for separation. The mobile phases consisted of 0.05% (v/v) formic acid in Milli-Q water (Phase A) and acetonitrile containing 0.05% (v/v) formic acid (Phase B). The gradient program was as follows: 0.0–1.0 min with 5% B; 1.0–2.0 min from 5–30% B; 2.0–14.0 min from 30–70% B; 14.0–15.0 min at 70–100% B; 15.0–16.0 min at 100% B; 16.0–17.0 min back to 5% B; and 17.0–20.0 min at 5% B. The mobile phase flow rate was set to 300 μL/min, and the column temperature was maintained at 30°C. The UHPLC system was coupled to a Compact micrOTOF-Q mass spectrometer (Bruker, Bremen, Germany) equipped with an electrospray ionization (ESI) source operating in negative ion mode. The ion spray voltage was set to -3900 V, with a dry gas temperature of 250°C and a flow rate of 8 L/min. Nitrogen was used as the dry gas, nebulizer gas, and collision gas, with a nebulizer pressure of 2.5 bar and a collision energy of 10 eV. MS spectra were collected in the m/z range of 50 to 1400, and MS/MS spectra were recorded from 200 to 1400 m/z, with a sampling rate of 3 Hz.


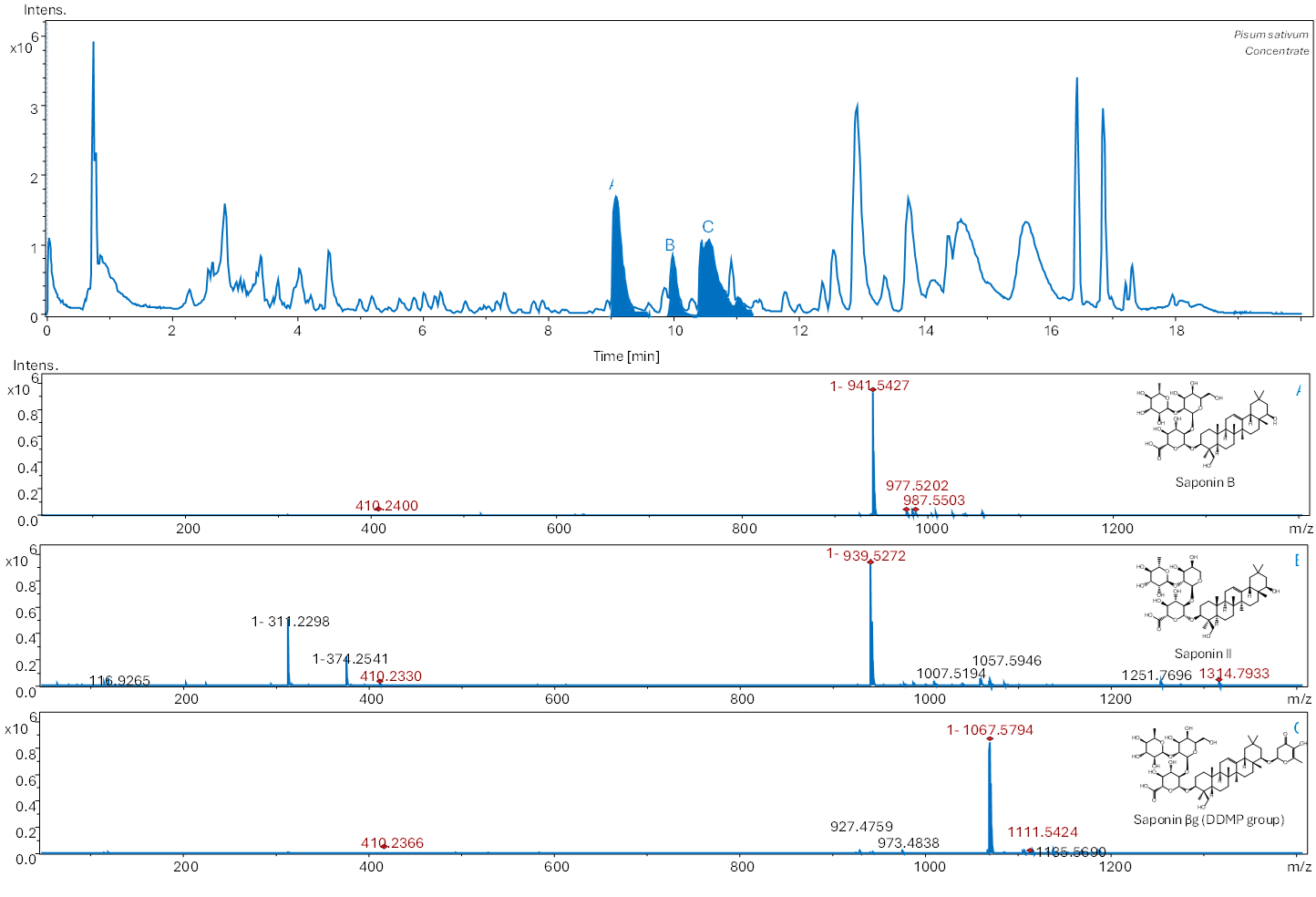


**Table S1.** Sensory vocabulary used for the evaluation of PP concentrates.

| Sensory attribute | Definition | Scale range | Reference material |
| --- | --- | --- | --- |
| Sweet | Taste associated with sucrose solution | none – a lot | Sucrose (12 g/L water) |
| Bitter | Taste associated with caffeine solution | a little – a lot | Caffeine (0.8 g/L water) |
| Umami | Taste associated with monosodium glutamate solution | none – a lot | Monosodium glutamate (1 g/L water) |
| Green pea | Flavour associated with fresh, raw, green peas - reminiscent of grass and vegetable | none – a lot | Fresh green peas and pea shoots |
| Yellow pea | Flavour associated with mild, earthy soaked/cooked yellow peas | none – a lot | Yellow split peas in hot water at 1:3 (w/v) |
| Nutty | Flavour associated with roasted peanuts, with a dry, toasted, slightly sweet note | none – a lot | Smooth peanut butter |
| Overall flavour intensity | Strength of all combined flavour notes perceived in the sample | weak – strong | None |
| Astringency | Dry, puckering sensation on the tongue and inner cheeks and gums | a little – a lot | Toasted white sandwich bread |
| Bitter aftertaste | Lingering bitterness remaining after swallowing/spitting, comparable to the aftertaste of black tea | a little – a lot | 2 black tea bags infused in 250 ml water |

**Table S2**. Protein content of the de-hulled peas.

| Cultivar | Akooma | Astronaute | Bagoo | Greenway | Ingrid | Kaplan |  | Karacter | Manager | Orchestra | Skol |
| --- | --- | --- | --- | --- | --- | --- | --- | --- | --- | --- | --- |
| Protein  Content (%) | 23.9 | 23.9 | 23.1 | 22.1 | 24.3 | 27.3 |  | 23.3 | 22.2 | 25.7 | 22.8 |

**Figure S1 and S2**. The results of the analysis of the PP ingredients’ physicochemical and foaming properties were subjected to a Pearson correlation analysis. The heatmap reveals a range of pair wise correlation coefficients, indicating both positive and negative relations between the data.

Abbreviations: FC-N foam capacity at natural pH, FC-6 foam capacity at pH 6, FC-7 foam capacity at pH 7, FC-8 foam capacity at pH 8, FS-N foam stability at natural pH, FS-6 foam stability at pH 6, FS-7 foam stability at pH 7, FS-8 foam stability at pH 8, S-N protein solubility at natural pH, S-4 protein solubility at pH 4, S-5 protein solubility at pH 5, S-6 protein solubility at pH 6, S-7 protein solubility at pH 7, S-8 protein solubility at pH 8, ZP-N ζ-potential at natural pH, ZP-4 ζ-potential at pH 4, ZP-5 ζ-potential at pH 5, ZP-6 ζ-potential at pH 6, ZP-7 ζ-potential at pH 7, ZP-8 ζ-potential at pH 8, D[3,2] surface average diameter, D[4,3] volume average diameter, PC protein content, L/V 11S/7S ratio, AA-P positive amino acids, AA-N negative amino acids, AA-T overall charge, TS total saponin, S-F soluble fiber, NS-F insoluble fiber, WHC water holding capacity, OHC oil holding capacity.

**Figure S1**. Heatmap of PP concentrate.


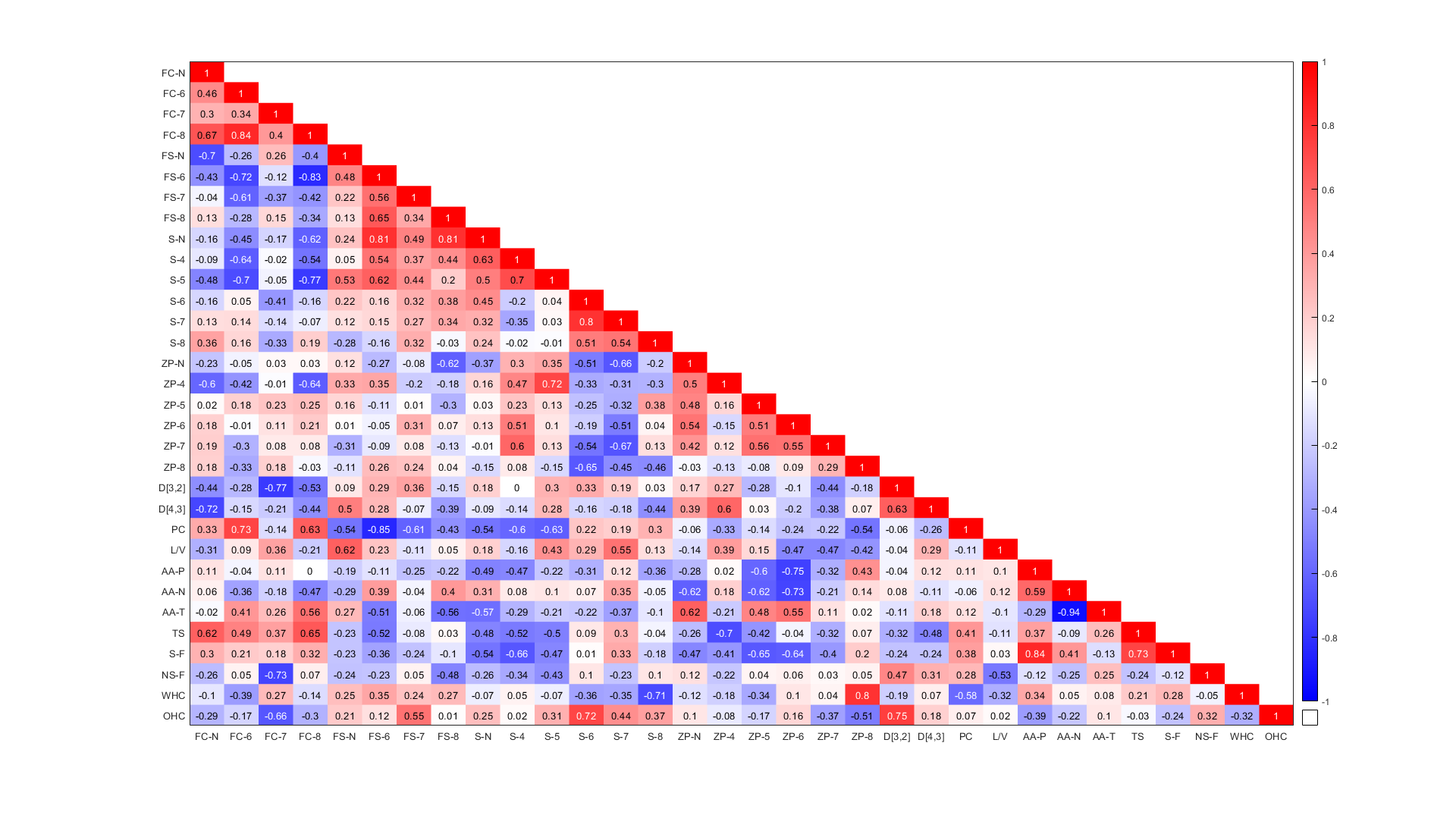


**Figure S2**. Heatmap of PP isolate.


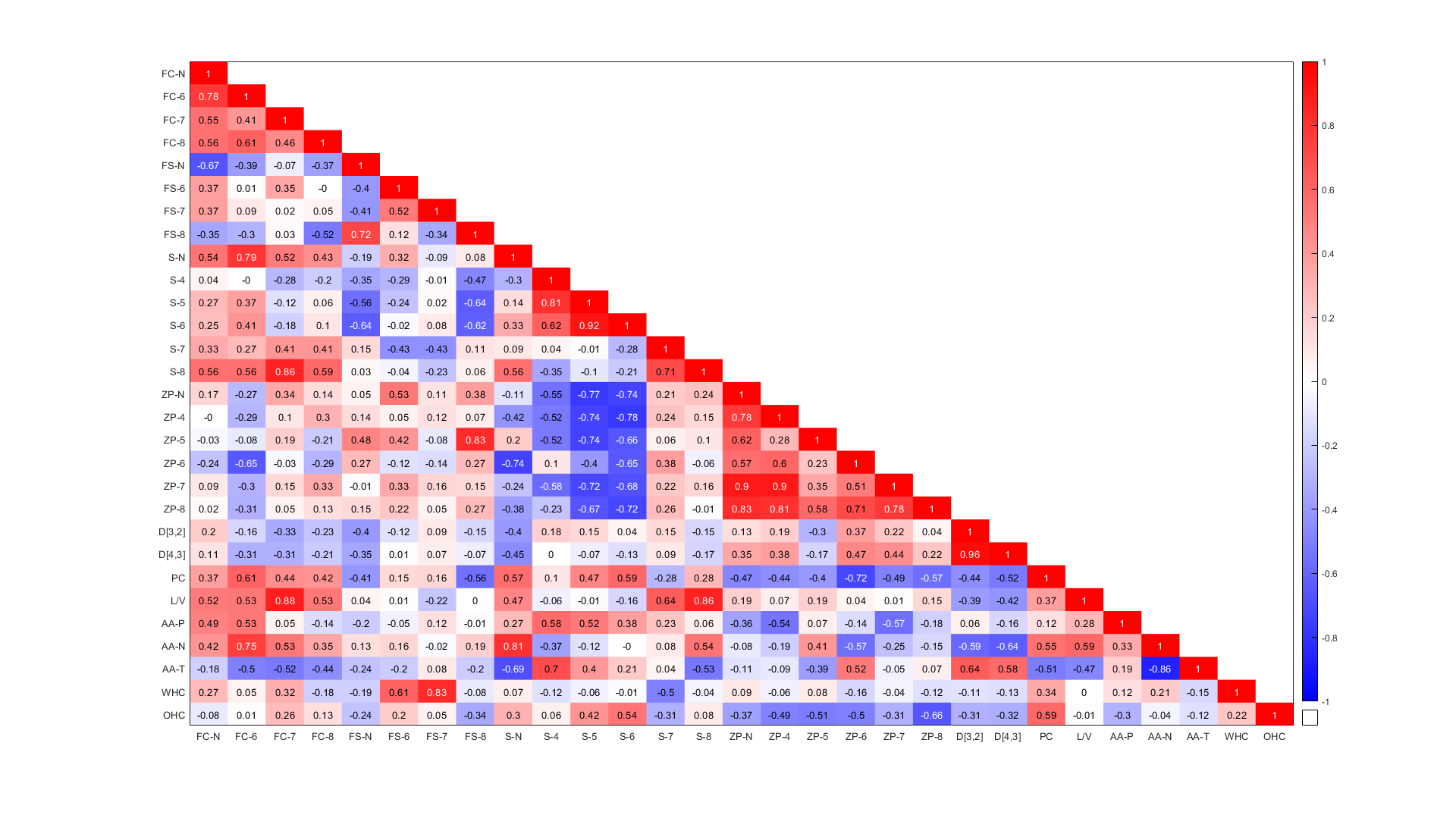

Supplement: Multimedia component 1 [file mmc1.docx]
